# Hemogram-Derived Inflammatory Indices and Metabolic-Renal Biomarkers as Predictors of Complications and Outcomes in Acute Cholecystitis

**DOI:** 10.7759/cureus.97946

**Published:** 2025-11-27

**Authors:** Admir Abdic, Emir Becirovic, Minela Becirovic, Amir Bećirović, Malik Ejubović, Elma Mujakovic, Amira Jagodić Ejubović

**Affiliations:** 1 Department of Surgery, Cantonal Hospital Bihać, Bihać, BIH; 2 Internal Medicine Clinic, Intensive Care Unit, University Clinical Center Tuzla, Tuzla, BIH; 3 Internal Medicine Clinic, Department of Nephrology, University Clinical Center Tuzla, Tuzla, BIH; 4 Department of Endocrinology and Internal Medicine, University Clinical Center Tuzla, Tuzla, BIH; 5 Department of Internal Medicine, Cantonal Hospital Zenica, Zenica, BIH; 6 Department of Anatomy, University of Tuzla, Tuzla, BIH

**Keywords:** acute cholecystitis, complications, creatinine, glucose, neutrophil-to-lymphocyte ratio, systemic immune-inflammation index

## Abstract

Background

Acute cholecystitis (AC) is one of the most common surgical emergencies with a wide range of clinical outcomes. Early identification of patients at risk for postoperative complications is essential for optimizing surgical decision-making and resource allocation. Hemogram-derived indices such as the systemic immune-inflammation index (SII) and neutrophil-to-lymphocyte ratio (NLR), in addition to biochemical markers, may provide prognostic value beyond traditional risk factors.

Materials and methods

This retrospective single-center study included 210 patients admitted to the University Clinical Center Tuzla with AC between January 2024 and January 2025. Demographic, clinical, and laboratory data were collected. Receiver operating characteristic (ROC) analysis was performed to identify optimal cut-off values for predicting complications. Multivariate logistic regression was adjusted for age, sex, diabetes mellitus, hypertension, and other baseline comorbidities, in addition to SII, NLR, glucose, and creatinine.

Results

Four variables emerged as independent predictors of complications: SII > 950 remained an independent predictor after full adjustment (p = 0.002) with a sensitivity of 78% and specificity of 72%. It yielded the highest discriminatory accuracy among the evaluated markers, with an area under the curve (AUC) of 0.81 (95% confidence interval (CI) 0.75-0.87). No formal comparison with TG18 grading was performed. In contrast, baseline comorbidities such as diabetes mellitus and hypertension did not retain significance after adjustment.

Conclusion

SII, NLR, glucose, and creatinine independently predicted complications in AC, with SII emerging as the strongest predictor among the evaluated variables. These findings suggest that incorporating hemogram-derived indices into preoperative assessment may enhance risk stratification. However, the retrospective single-center design and potential confounding related to the surgical approach warrant cautious interpretation.

## Introduction

Acute cholecystitis (AC) is among the most frequent abdominal surgery emergencies and continues to represent a significant burden for healthcare systems worldwide [1]. Gallstone disease is the leading cause, affecting 10%-20% of adults. Sometimes, the gallstones obstruct the cystic duct, leading to gallbladder distension, inflammation, and a secondary bacterial infection. If untreated, AC may progress to severe complications such as gangrene, perforation, or sepsis, all associated with high morbidity and mortality [2,3]. AC is one of the leading indications for emergency cholecystectomy, a lifesaving procedure. Patients usually present with right-upper-quadrant pain, fever, nausea or vomiting, and a positive Murphy’s sign [4]. Laboratory results often reveal leukocytosis and elevated inflammatory markers, while mild hyperbilirubinemia may indicate biliary obstruction. The incidence is higher in women and older adults, populations that usually carry additional comorbidities that may contribute to worse outcomes [5].

The Tokyo Guidelines 2018 (TG18) provide standardized diagnostic criteria and severity grading into mild, moderate, and severe forms, guiding therapeutic approaches from early laparoscopic cholecystectomy to delayed surgery or percutaneous drainage in critically ill patients [6]. Although TG18 severity grading provides a structured clinical framework, several observational studies have reported variability in its ability to anticipate postoperative complications, suggesting that supplementary markers may improve early risk stratification [7].

Traditional laboratory markers such as C-reactive protein (CRP), white blood cell (WBC) count, and total bilirubin (TBil) have been associated with complicated AC, but their predictive capacity is limited. More recently, hemogram-derived indices have gained attention as integrative markers of systemic inflammation and immune response [8]. In AC, the inflammatory cascade leads to the activation of neutrophils, suppression of lymphocytes, and mobilization of platelets. These changes are captured by ratios such as the neutrophil-to-lymphocyte ratio (NLR) and platelet-to-lymphocyte ratio (PLR). More comprehensive indices, including the systemic immune-inflammation index (SII) and pan-immune-inflammation value (PIV), combine these cell counts to reflect both the intensity and balance of the host’s inflammatory reaction [9]. Additionally, metabolic and renal biomarkers such as serum glucose, creatinine, and urea may further refine risk assessment, reflecting the systemic stress response and early organ dysfunction that can accompany severe inflammation in AC [10]. This retrospective study was, therefore, designed to investigate whether hemogram-derived inflammatory indices and simple metabolic-renal biomarkers obtained at admission could serve as predictors of complications in patients undergoing cholecystectomy for AC.

## Materials and methods

This single-center retrospective study was conducted at the Department of General and Abdominal Surgery, University Clinical Center Tuzla, Bosnia and Herzegovina. All consecutive adult patients who underwent cholecystectomy for AC between January 1, 2024, and January 31, 2025, were included in the study. The diagnosis of AC was established according to the TG18 [11]. Patients with chronic cholecystitis, gallbladder malignancy, or incomplete medical records were excluded.

Clinical and operative data were extracted from electronic medical records, including demographic variables, comorbidities, clinical presentation, TG18 severity grade, admission laboratory results, operative details, complications, and length of hospital stay (LOS). Complications were defined as acute cholangitis, pancreatitis, gallbladder perforation, empyema, gangrenous cholecystitis, or sepsis during hospitalization. This composite outcome reflects both advanced disease at presentation and complications arising during the hospital course, serving as a proxy for a severe or complicated clinical trajectory. Patients were stratified into two groups: those with uncomplicated AC and those with complicated AC.

Venous blood samples were obtained at admission, before the initiation of therapy. Complete blood count was performed using the Sysmex XN-1000 automated hematology analyzer (Sysmex Corporation, Kobe, Japan). Biochemical parameters, including glucose, urea, creatinine, and TBil, were measured with the Beckman Coulter DxC 700 AU chemistry analyzer (Beckman Coulter Diagnostics, Brea, CA, USA). From hematological parameters, the following indices were calculated: NLR = neutrophils/lymphocytes; PLR = platelets/lymphocytes; SII = neutrophils × platelets/lymphocytes; PIV = neutrophils × platelets × monocytes/lymphocytes. All indices were derived using absolute cell counts obtained from the complete blood count at admission.

Operative findings were recorded immediately postoperatively and included the surgical approach (laparoscopic, open, or conversion), the urgency of the procedure (emergency vs. elective), and the presence of complications such as gangrenous, perforated, or fistulous gallbladders, empyema, abscess formation, or peritonitis. The primary outcome of interest was the development of in-hospital complications. LOS was analyzed as a secondary outcome, categorized into ≤7 days and >7 days according to the cohort’s median value.

The study was approved by the Ethics Committee of the University Clinical Center Tuzla (Approval No: 02-09/2-208-3/24). Owing to the retrospective design and anonymized dataset, informed consent was waived. All data were stored on a password-protected institutional server in compliance with institutional and national data protection standards.

Statistical analysis

Statistical analyses were performed using Jamovi software, version 2.6 (The Jamovi project, 2021, https://www.jamovi.org). The distribution of continuous variables was examined using the Shapiro-Wilk test. As most variables deviated from a normal distribution, non-parametric methods were applied. Continuous variables are presented as medians with interquartile ranges (IQRs), while categorical variables are expressed as frequencies and percentages.

Comparisons between patients with complicated and uncomplicated AC were performed using the Mann-Whitney U test for continuous variables and Pearson’s chi-squared test for categorical variables. Receiver operating characteristic (ROC) analysis was conducted to evaluate the predictive ability of hematological indices (NLR, PLR, SII, and PIV) and biochemical parameters (glucose, urea, and creatinine) for complications. Areas under the curve (AUCs) with 95% confidence intervals (CIs) were calculated, and optimal cut-off values were determined using the Youden index. Pairwise comparisons of AUC values were performed using DeLong’s test to assess statistical differences in discriminatory capacity.

Variables significant in univariate analyses (p < 0.10) were entered into a multivariate logistic regression model to identify independent predictors of complications. Predictors were dichotomized according to ROC-derived thresholds to facilitate clinical applicability. Model calibration was assessed with the Hosmer-Lemeshow goodness-of-fit test, while the AUC of the final model quantified discriminative ability. All statistical tests were two-tailed, and statistical significance was defined as p < 0.05.

## Results

A total of 210 patients with AC were analyzed, of whom 118 (56.2%) had an uncomplicated clinical course and 92 (43.8%) developed complications. Patients with complicated AC were significantly older (67.8 ± 12.5 vs. 61.4 ± 13.2 years; t = 2.91, p = 0.004). Although male sex (51/92, 55.4% vs. 62/118, 52.5%), diabetes mellitus (32/92, 34.8% vs. 27/118, 22.9%), and hypertension (57/92, 62.0% vs. 59/118, 50.0%) were more frequent in the complicated group, these differences did not reach statistical significance (all p > 0.05). Hospitalization duration was significantly longer in patients with complications (8.6 ± 3.2 vs. 5.1 ± 2.4 days; t = 8.22, p < 0.001).

Inflammatory and hematologic parameters showed apparent differences between groups. WBC counts and neutrophil counts were significantly higher in complicated cases (U = 3,105.5, both p < 0.001), whereas lymphocyte counts were lower (U = 2,854.0, p < 0.001). Platelet counts were modestly increased (298 (248-342) vs. 265 (220-312) ×10⁹/L; U = 4,721.0, p = 0.02). Consequently, all hemogram-derived indices, including NLR, PLR, SII, and PIV, were consistently and significantly higher in the complicated group (all p < 0.001). Glucose, urea, and creatinine were likewise significantly elevated in the complex group (all p < 0.001) (Table 1).

**Table 1:** Baseline demographic, clinical, and laboratory characteristics of patients with AC according to complication status Data are presented as mean ± SD, median (IQR), or n (%), as appropriate. p-values were calculated using Student’s t-test (continuous normally distributed variables), Mann-Whitney U test (continuous non-normally distributed variables), or the chi-squared test (categorical variables), as applicable. A p-value < 0.05 was considered statistically significant. AC: acute cholecystitis; SD: standard deviation; WBC: white blood cell; IQR: interquartile range; NLR: neutrophil-to-lymphocyte ratio; PLR: platelet-to-lymphocyte ratio; SII: systemic immune-inflammation index; PIV: pan-immune-inflammation value.

Variable	Uncomplicated AC (n = 118)	Complicated AC (n = 92)	Test statistic	p-value
Age (years) (mean ± SD)	61.4 ± 13.2	67.8 ± 12.5	t = 2.91	0.004
Male sex, n (%)	62 (52.5)	51 (55.4)	χ² = 0.17	0.68
Diabetes mellitus, n (%)	27 (22.9)	32 (34.8)	χ² = 3.29	0.07
Hypertension, n (%)	59 (50.0)	57 (62.0)	χ² = 2.84	0.09
WBC (×10⁹/L) (median (IQR))	10.8 (8.9–12.6)	13.2 (11.4–15.5)	U = 3,105.5	<0.001
Neutrophils (×10⁹/L)	7.2 (6.0–9.1)	10.1 (8.5–12.0)	U = 3,105.5	<0.001
Lymphocytes (×10⁹/L)	2.2 (1.7–2.6)	1.5 (1.1–2.0)	U = 2,854.0	<0.001
Platelets (×10⁹/L)	265 (220–312)	298 (248–342)	U = 4,721.0	0.02
Glucose (mmol/L)	5.9 (5.1–6.7)	7.3 (6.2–8.9)	U = 2,888.0	<0.001
Urea (mmol/L)	5.4 (4.3–6.6)	7.1 (5.8–8.4)	U = 3,310.0	<0.001
Creatinine (µmol/L)	79 (68–93)	98 (83–122)	U = 3,261.0	<0.001
NLR	3.4 (2.6–4.8)	7.2 (5.3–9.0)	U = 2,490.5	<0.001
PLR	124 (101–148)	168 (139–202)	U = 2,550.0	<0.001
SII	710 (520–880)	1,180 (960–1,420)	U = 2,330.0	<0.001
PIV	285 (230–340)	420 (350–500)	U = 2,415.0	<0.001

ROC curve analysis demonstrated that hemogram-derived inflammatory indices had superior predictive performance compared with biochemical markers. The SII achieved the highest discriminatory ability (AUC = 0.81, 95% CI 0.75-0.87), followed by NLR (AUC = 0.79) and PIV (AUC = 0.77). PLR showed moderate predictive capacity (AUC = 0.74). Among metabolic-renal biomarkers, creatinine (AUC = 0.73) and glucose (AUC = 0.72) performed better than urea (AUC = 0.70). By DeLong’s test, SII significantly outperformed glucose and urea (p < 0.05). All AUC values were statistically significant (p < 0.01) (Table 2). Optimal cut-off values identified by ROC analysis (SII > 950, NLR > 4.5, glucose > 6.8 mmol/L, and creatinine > 90 μmol/L) matched the thresholds retained in the multivariate model, reinforcing their clinical interpretability.

**Table 2:** ROC analysis of hemogram-derived indices and metabolic-renal biomarkers for the prediction of complications in acute cholecystitis Data are presented as AUC with 95% CIs, optimal cut-off values, sensitivity, and specificity derived from receiver operating characteristic (ROC) analysis. p-values were calculated using DeLong’s test for comparison of AUCs. A p-value < 0.05 was considered statistically significant. AUC: area under the curve; CI: confidence interval; NLR: neutrophil-to-lymphocyte ratio; PLR: platelet-to-lymphocyte ratio; SII: systemic immune-inflammation index; PIV: pan-immune-inflammation value.

Biomarker	AUC (95% CI)	Cut-off value	Sensitivity (%)	Specificity (%)	p-value
NLR	0.79 (0.72–0.85)	>4.5	76	71	<0.001
PLR	0.74 (0.66–0.81)	>150	70	68	<0.001
SII	0.81 (0.75–0.87)	>950	78	73	<0.001
PIV	0.77 (0.70–0.84)	>350	72	70	<0.001
Glucose	0.72 (0.65–0.79)	>6.8 mmol/L	68	65	<0.001
Urea	0.70 (0.62–0.77)	>6.5 mmol/L	65	64	<0.01
Creatinine	0.73 (0.66–0.80)	>90 µmol/L	69	66	<0.001

On univariate analysis, SII, NLR, creatinine, and glucose were significantly associated with complications (all p < 0.01), whereas age, sex, diabetes mellitus, and hypertension did not reach statistical significance. In the multivariate analysis, four independent predictors of complications remained significant: SII > 950 (odds ratio (OR) 2.45, 95% CI 1.40-4.30, p = 0.002), creatinine > 90 μmol/L (OR 2.12, 95% CI 1.20-3.76, p = 0.008), NLR > 4.5 (OR 1.98, 95% CI 1.15-3.42, p = 0.014), and glucose > 6.8 mmol/L (OR 1.87, 95% CI 1.05-3.32, p = 0.031). Neither diabetes mellitus nor hypertension retained significance. The final model demonstrated good calibration (Hosmer-Lemeshow p = 0.47) and strong discriminative performance (AUC = 0.83, 95% CI 0.77-0.88) (Table 3).

**Table 3:** Multivariate logistic regression analysis for the prediction of complications in patients with acute cholecystitis Data are presented as ORs with 95% CIs from multivariate logistic regression. p-values were calculated using the Wald χ² test. A p-value < 0.05 was considered statistically significant. OR: odds ratio; CI: confidence interval; SII: systemic immune-inflammation index; NLR: neutrophil-to-lymphocyte ratio.

Variable	OR (95% CI)	Wald χ²	p-value
SII > 950	2.45 (1.40–4.30)	9.41	0.002
NLR > 4.5	1.98 (1.15–3.42)	5.98	0.014
Creatinine > 90 µmol/L	2.12 (1.20–3.76)	7.10	0.008
Glucose > 6.8 mmol/L	1.87 (1.05–3.32)	4.63	0.031

**Figure 1:**
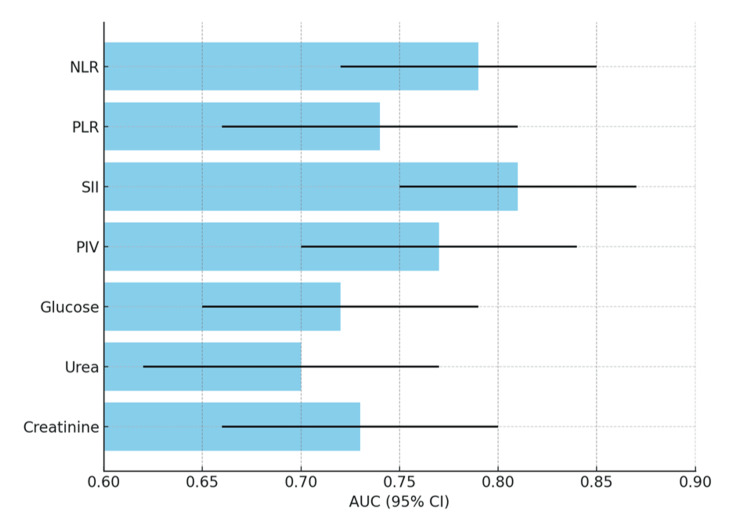
Summary of AUC values (95% CI) derived from ROC analyses for predicting complications in acute cholecystitis Bars represent the AUC values with 95% CIs for each biomarker. The figure summarizes results obtained from ROC analyses but does not display ROC curves themselves. This figure is intentionally presented as an AUC comparison plot rather than a traditional ROC curve plot, to allow clearer visualization and comparison of discriminative performance across biomarkers. p-values for AUC comparisons were calculated using the DeLong test, with significance defined as p < 0.05. The SII demonstrated the highest discriminative performance (AUC 0.81), followed by the NLR (0.79), PLR (0.74), and PIV (0.77). Among biochemical markers, creatinine (0.73) and glucose (0.72) showed moderate ability, whereas urea had the lowest discriminative value (0.70). AUC: area under the curve; CI: confidence interval; NLR: neutrophil-to-lymphocyte ratio; PLR: platelet-to-lymphocyte ratio; SII: systemic immune-inflammation index; PIV: pan-immune-inflammation value; ROC: receiver operating characteristic.

The discriminative ability of hemogram-derived indices and biochemical markers is illustrated in the AUC comparison plot summarizing ROC analysis. SII provided the highest prognostic accuracy, outperforming other indices and biochemical markers, underscoring its role in identifying patients at risk of complicated AC (Figure 1).

**Figure 2:**
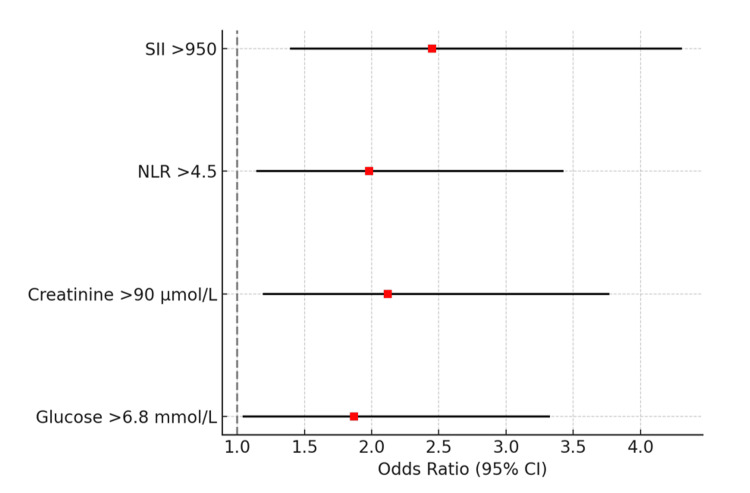
Forest plot of independent predictors of complications in acute cholecystitis The forest plot shows ORs with corresponding 95% CIs for independent predictors of complications derived from multivariate logistic regression analysis. p-values were calculated using the Wald χ² test, with statistical significance defined as p < 0.05. SII (>950), NLR (>4.5), creatinine > 90 μmol/L, and glucose > 6.8 mmol/L remained significant predictors of complicated acute cholecystitis, with CIs not crossing unity. OR: odds ratio; CI: confidence interval; SII: systemic immune-inflammation index; NLR: neutrophil-to-lymphocyte ratio.

The forest plot shows that SII > 950, NLR > 4.5, creatinine > 90 μmol/L, and glucose > 6.8 mmol/L all remained independent predictors, with ORs exceeding 1.0 and CIs not crossing unity (Figure 2).

## Discussion

In this analysis of 210 patients with AC, we identified four independent predictors of complications: SII, NLR, serum creatinine, and glucose levels. Among these, SII demonstrated the strongest discriminatory performance, clearly outperforming both traditional biochemical markers and other hemogram-derived indices. These findings highlight the central role of systemic inflammatory burden, together with metabolic-renal dysfunction, in shaping the clinical course of AC.

Our results are consistent with the growing evidence that hemogram-derived indices provide superior prognostic information compared to single inflammatory markers. Both SII and NLR reflect the balance between neutrophil-driven inflammation and lymphocyte suppression, thereby integrating innate and adaptive immune responses [12]. The robust predictive capacity of SII observed in our cohort suggests it may serve as a reliable surrogate for systemic immune activation, complementing clinical and imaging findings in preoperative assessment [13]. Similar findings have been reported in studies of gastrointestinal and hepatobiliary emergencies, where SII and NLR outperformed traditional markers in predicting complications and adverse outcomes [14]. Comparable associations have been reported in other acute inflammatory conditions, including specific cardiovascular presentations. However, these parallels primarily support the concept that hemogram-derived indices are general markers of systemic immune activation rather than imply direct extrapolation to AC [15].

The associations of hyperglycemia and elevated creatinine with complicated outcomes emphasize the vulnerability of patients with metabolic and renal dysfunction. Hyperglycemia may reflect both stress-induced glucose dysregulation and pre-existing impaired glycemic control, each of which amplifies inflammatory pathways and reduces host defense capacity [16]. Elevated creatinine, on the other hand, likely captures a combination of underlying chronic kidney disease, systemic inflammation, and acute illness-related renal dysfunction, all of which predispose to adverse outcomes [17]. This aligns with prior evidence linking renal impairment and perioperative hyperglycemia to worse outcomes in abdominal surgery [18].

Interestingly, traditional risk factors such as diabetes mellitus and hypertension did not retain independent significance after adjustment. This suggests that acute inflammatory and metabolic derangements exert more substantial short-term influence on complication risk. However, interactions with baseline comorbidities, particularly the relationship between diabetes and stress hyperglycemia, may still contribute to individual variability. The fact that admission glucose remained a significant predictor while diabetes itself did not highlights the prognostic importance of acute physiological derangement, reinforcing the notion that the immediate metabolic response to illness carries greater short-term relevance than the chronic diagnostic label. Such findings support the concept that dynamic biomarkers of the acute phase provide more accurate prognostic insight than baseline comorbidity profiles alone [19,20].

From a clinical perspective, the use of cut-off values identified by ROC analysis (SII > 950, NLR > 4.5, glucose > 6.8 mmol/L, and creatinine > 90 µmol/L) provides a practical framework for early risk stratification. These thresholds may help clinicians identify high-risk patients upon admission, prioritize surgical timing, and tailor perioperative care accordingly. The integration of such markers into routine evaluation could be particularly valuable in resource-limited settings, where rapid and cost-effective tools for risk stratification are urgently needed [21].

Limitations

This study has several limitations. It was conducted at a single tertiary center, which may limit its generalizability. The observational design cannot exclude residual confounding, despite multivariate adjustment. We did not stratify intraoperative findings and postoperative complications in detail, which may have provided additional insights. Moreover, long-term outcomes such as readmissions or mortality were not assessed. Another limitation is the absence of external validation, which should be addressed in future multicenter prospective studies.

## Conclusions

Our study demonstrates that SII, NLR, creatinine, and glucose are independent predictors of complications in AC, with SII emerging as the most powerful marker. The integration of hemogram-derived indices and simple biochemical parameters into preoperative evaluation may improve early risk stratification and guide surgical decision-making. These results underscore the importance of systemic inflammation and metabolic-renal dysfunction in the pathogenesis of complicated disease and highlight their potential role in optimizing hospital resource utilization.
